# Perspectives for the Use of Social Media in e-Pharmamarketing

**DOI:** 10.3389/fphar.2016.00445

**Published:** 2016-11-21

**Authors:** Magdalena Syrkiewicz-Świtała, Piotr Romaniuk, Ewa Ptak

**Affiliations:** ^1^Department of Health Economics and Health Management, School of Public Health in Bytom, Medical University of Silesia in KatowiceKatowice, Poland; ^2^Department of Health Policy, School of Public Health in Bytom, Medical University of Silesia in KatowiceKatowice, Poland

**Keywords:** social media marketing, social media, pharmamarketing, health management, public health

## Abstract

The Internet has become a common and global medium. It contains vast information resources and a wide range of communication instruments. One of the communication channels are social media, which increasingly are also used in the business field. Social media combined with mobile technology introduced new challenges to marketing activity. This trend is also observed on specific and highly regulated drug market. The aim of this article is to describe the perspectives for the use of social media in e-pharmamarketing. We find that this require personalized communication, the use of online promotion tools, in order to perform advertising in contact with a demanding client. Currently pharmaceutical companies use social media to communicate basic information on their life, but they still do not appreciate it sufficiently as a tool to build the image of company or products. It is therefore considered that these companies should attach greater importance to the presence in this type of media, especially in the light of dynamic changes in the way people communicate.

## Introduction

The Internet has revolutionized the world and is growing rapidly, the same as possibilities of using it in the functioning of society and business. Better access to the global network increases the number of users [46% of world population – 3,42 billion people (Blog smmeasure. Liczby Polskiego Internetu, 2016)]. The traditional media are being replaced by the Internet to some extent, because of its accessibility: convenient time of information releases and the possibility of selecting interesting news by the recipient. One of the online media is social media, which is currently used by 2.31 billion people in the world (Mobirank, 2016), including 1.97 billion (27% of the world’s population) using mobile devices. (Mobirank, 2016). Their main advantage is the interactivity and two-way flow of information (Syrkiewicz-Świtała and Świtała, 2012).

Since the introduction of e-mail, new media (e.g., YouTube) and social networking (e.g., Facebook, and Twitter) the ways of human communication have changed noticeable. These changes are observed regardless of the age of Internet users. This is obviously more popular among the younger generation but it is used increasingly among the elderly as well (Syrkiewicz-Świtała, 2015b). Popular nowadays social media, which were meant only to maintain ties and relationships, has also become a place to promote and strengthen the image of companies (Chaudhry, 2011; Gibson, 2012; Królewski and Sala, 2014). They have created a new paradigm of communication, which is based on web surfers’ activity. Internet users make and distribute Internet transmission, they become even prosumers (Pikuła-Małachowska, 2010; Syrkiewicz-Świtała, 2014). These new media outdated existing action scheme in marketing. In last 5 years, the importance of social media has increased significantly, and market activities in this area are the fastest growing sector of Internet marketing. Social media become finally also a source of health information (Evans, 2006; Bernhardt et al., 2014; Syrkiewicz-Świtała and Świtała, 2015). In recent years, Facebook has become one of the most popular sources of information on health in the UK (Collier, 2014; Syrkiewicz-Świtała, 2015a).

The use of modern technology in the pharmaceutical market is not only the implementation of modern production techniques and supply of consumers with innovative medicinal products. The dynamic development of interactive electronic media of a social network gives also a huge field of possibilities for the use of innovative methods of selling and building market position of companies operating in the pharmaceutical industry. Thus is created e-pharmamarketing. It is an activity of a pharmaceutical company aimed to promote and sell its products and build a relationship with the customer or selected groups of recipients, conducted via the Internet (Armstrong and Kotler, 2012). The purpose of this article is to describe the prospects for the use of e-pharmamarketing via social media.

## The Experience of Pharmaceutical Companies in Social Media Marketing

Marketing of pharmaceutical companies in social media can rely on generating Internet traffic by the use of social networking sites. It enables direct contact with a target group, also reducing marketing costs. It aims at formation of relationship between a pharmaceutical company and doctors or patients. This is not a method to achieve rapid sales growth, but it is to become a partner for dialog. The goal is to influence positive image of a company and, at the same time, to encourage customers to share information with friends. Social media marketing relies on effective fanpage management of a brand or a company. This is public relations and marketing operation. Pharmaceutical companies may place counseling articles on social networking sites. The topics may cover health, medicine, lifestyle, nutrition, etc. Photos, videos, commercials, and short text messages can be also published.

The authors of this article conducted a survey on 50 biggest pharmaceutical companies operating in Poland (Pharmaceutical Representative luty-marzec 2014/nr1(31) TOP 50, 2016). The obtained results showed that the vast majority of pharmaceutical companies (86% of companies surveyed) has an account on a social networking site. The most popular services were: Facebook (76% of responses), Twitter (34%), LinkedIn (32%) and Google+ (12%). These are also the most popular portals globally. Facebook unites 1.59 billion users worldwide (Blog smmeasure. Liczby Polskiego Internetu, 2016), Twitter 320 million users (Blog smmeasure. Liczby Polskiego Internetu, 2016), LinkedIn has a base of 100 million people (Blog smmeasure. Liczby Polskiego Internetu, 2016), and Google+ although is the youngest (opened in 2011) was able to convince already 1 billion Internet users (Grabiec, 2016).

A vast majority of pharmaceutical companies benefit from the YouTube platform as well. YT has already over 1 billion users in the world (YouTube Statystyki, 2016). 70% of surveyed companies provide audiovisual messages in this way, but only 32% of them declare the use of YT for advertising purposes. This may be due to legal restrictions on advertising of medicines in different countries. The companies use YT to present their functioning (company events, charitable activities, drug manufacturing process and other information that can positively affect the company’s image). Thanks the dynamic ability to distribute similar types of materials in social networks, companies gain maximum reach and brand visibility. It is done with a relatively small outlay of work by PR and marketing professionals.

Video marketing and discussion forums are much less used. Blogging is not popular. None of companies used chat. While social networking, the companies: provide information reference (56%), try to build the company image (54%), communicate professional staff and consumer (28%). 26% of surveyed companies want to increase sales of their products. Only few of them (8%) use social media to strengthen brand awareness.

Unfortunately, microblogs are still less popular than social networking among pharmaceutical companies. Microblogs provide the opportunity for pharmaceutical companies to submit short entries, slogan, logo, links to other reference sites or articles. Microblog’s entries are only to encouraged to click on a link and read full content on a website or enter into a discussion. The companies often indicate briefly on a received award, sponsored events, study on a new product, a new drug registration and others.

## Perspectives for the Use of E-Pharmamarketing Carried out through Social Media

E-pharmamarketing in social media is not without disadvantages. Among the most important are the lack of immediate results and difficult measurability. It is because similar types of activities are carried out in an integrated promotional activities of pharmaceutical companies (Düssel, 2009; Heuristic, 2016). It is also a time-consuming form which requires regularity, driving continual dialog, permanent relationships maintaining and involvement (Heuristic, 2016). This form is insufficient in developing countries, where the problem of digital exclusion is widespread. Even among EU member states this is not completely abstract problems. In Romania less than 50% of the population use the Internet (Wykluczenie cyfrowe w Polsce. Raport grudzieñ, 2015).

Not without significance is the lack of full acceptance of social media. There is relatively low confidence in the network and the Internet users dislike impudence (spam; Hamala, 2014; Heuristic, 2016). The overabundance of information available on the Internet raises the noise effect in communication and searching for true and accurate content (Syrkiewicz-Świtała and Świtała, 2012). No full control over the flow of information provides the risk of unauthorized content, as well as an unethical actions – information fraud, falsification of identity or privacy violation (Hamala, 2014; Heuristic, 2016).

A significant difficulty is in final subordination of distributed content to legal and ethical requirements in different countries and cultures, which reduces the possibility of full universalization of globally distributed content.

Nevertheless, there are a lot of potential benefits: high interactivity, knowing competition and customer expectations (Syrkiewicz-Świtała and Świtała, 2015), possibility of far-reaching personalization of media and communication (Nowy Marketing, 2015b; Raport IRCenter.com styczeñ, 2016), expanding the availability of online communication channels (Google Trends, 2016), high dynamic of communication spread (Nowy Marketing, 2015b) and possibility of building a positive company image (Szydłowska, 2013; RAPORT, 2016). In the light of the observable trends it is simply necessary to maintain a competitive position in the market.

The potential of social media, however, still seems to be overlooked by pharmaceutical companies (Ustawa, 2001; Niedzielska and Syrkiewicz-Świtała, 2011; Syrkiewicz-Świtała et al., 2015). Many companies neglect important aspects of the use of tools such as e.g., ability to know the expectations of customers, as well as free publicity (*n* = 2; 4%) and the ability to control competition. As burdensome is perceived the need to constantly monitor the content or possibility of losing the positive company image, as well as lack of control over the content being placed. As a result, companies operating in the pharmaceutical industry tend to remain at least one step behind the technological progress in the field of communication tools, losing the ability to generate available benefits. One example of this may be Polish pharmacies. According to conducted monitoring in social media by the Institute of Media Monitoring (IMM) in the first half of 2016 only 20% requests for pharmacy recommendation received answers within the first 36 h after publication. So 80% of pharmacies lost the possibility of contact with the client in the network (Marketing, 2016). Companies do not appreciate the potential of specialized portals such as GoldenLine, which is the local equivalent of LinkedIn, visited by almost 2 million Internet users in Poland (Wirtualnemedia.pl., 2016). Potentially, this is a very useful tool in the process of the recruitment of skilled workers (Kowalczyk, 2012; Profil firmy Adamed na portalu LinkedIn, 2016).

It is expected to boost the activity of companies in this field. It is inevitable for the substantial development of modern mobile technologies, which directly translates to the popularity of social media. However, one can expect the change of interest of Internet users in specific digital medium. Perhaps, instead of popular Facebook there will be some other medium more attractive for customers. Therefore, it is important to monitor constantly the activities of Internet users in social media and on specific portals. Technological progress creates new marketing opportunities for pharmaceutical companies. It also forces to follow new trends, or their creation. Companies need to take creative and innovative approach and to act beyond tamed schemes.

## Conclusion

Skillfully lead social media pharmamarketing can help build a good relationship with the client on a specific and highly regulated pharmaceutical market. It can be helpful in communicating all necessary information about pharmaceutical products but also promote pro-health actions in the field of promotion and prevention (Akhtar et al., 2015). Social media marketing of pharmaceutical companies is therefore a growing area with great potential. This development is also supported by certain organizational measures on the drug market (Holecki et al., 2013). In November 2015, American Food and Drug Administration (FDA) has encouraged the use of social media to improve communication and information exchange in health promotion and public health (U.S. Food and Drug Administration Social Media Policy, 2015). Foreign studies show that one in four interactions with doctor, patient, and healthcare provider in the United States is a digital contact (Social media in the pharmaceutical sector. UK Synapse, 2013). Patient education through social media is therefore an opportunity for the pharmaceutical industry to gain confidence in the company and increase the awareness of consumer when choosing a product. In this way, customer acquires knowledge about health, diseases, and treatment. In various social media channels it is possible to find information on any drug. This information is available on: websites of a manufacturer, social network brand fanpages, portals for white staff specialists. According to a study, conducted by Comscore, patients who are familiar with drug brand website often followed the recommendations for its use (20% of patients). Internet advertising also influenced the use of a drug (13.5% of patients; ROI Media, 2016). E-pharmamarketing activities in social media and in the network tend to increase. It is estimated that in the year 2016 the US pharmaceutical companies allocate for this purpose 2.48 billion dollars (Berezowski, 2015). Media house Codemedia reported that spending on advertising in Poland on the Internet reaches 7%, i.e., more than 17 million PLN (year 2015) and it increased by 68%, as compared to the previous year (Nowy Marketing, 2015a).

Having a website is no longer a novelty by pharmaceutical companies, but actions in social media are innovative. It is still a very demanding area, obliging a good knowledge of the medical community and the needs of patients, as well as the compliance of pharmaceutical law in force in a country. All information on the websites of drug manufacturers is assessed by regulatory agencies in terms of their compliance with the pharmaceutical law. Therefore, besides the promotion of a brand itself, much of the information applies to illnesses, possible treatments, preventions, health information and healthy lifestyles (Bell et al., 2014). By placing the counseling health related topics, the public awareness increases. Thus positive recommendations among Internet users translate into sales (Akhtar et al., 2015). Every advertising or educational campaign carried out in social media must be very well thought out. It requires also constant monitoring of various social media. It should be also noted that the conduct of strategy in social media cannot be standardized, despite the fact that most companies have a global reach. It is to mention that widespread use of social media is very similar in all markets but the reactions of people belonging to different cultures are very different. Therefore, companies in this marketing strategy in social media should use adaptive approach i.e., the content and form of communication must comply with the specifics culture of the community. Then that action in this field will be more effective. Involvement in social media will help to build a positive relationship oriented to the needs of a particular client.

Activities in social media may be helpful also in emergency situations. They may assist in all situations such as: pollution in batches of the drug or an incorrect description on the packaging. In such situation it is important to reach a wide audience quickly and effectively. Social media can supplement the instruments used in effective crisis management. They can be used as a source of transmission of reliable and proven information to prevent dissemination of adverse scheme: no information – guess – rumors. Conducting marketing strategies of pharmaceutical companies through social media has also a social significance. It is also possible to use social media in emergency situations i.e., natural disasters (floods, earthquakes, etc.). Most of the large pharmaceutical companies, operating on corporate principles, are involved yet in corporate social responsibility (CSR) and for this purpose they reserve certain budgetary measures. By communicating via social media, companies can rapidly engage the local community for various rescue operations, supporting them financially and materially. Through such activities they gain synergy and their aid is effective and not anonymous. Therefore, companies can expect positive effects in the dimension of public relations.

Pharmaceutical companies can achieve many different goals through the use of social media. They can improve the level of knowledge; build relationships; increase sales of its own products and finally serve general social purposes. As they seem not to take the possible advantages arising of e-pharmamarketing yet, it should be recommended they clearly re-orientate their marketing activity toward new instruments of communication available via Internet to stay up to date with the dynamic changes appearing in the social life and communication channels.

## Author Contributions

MS-Ś conceived the study and prepared draft of the paper. MS-Ś and PR contributed to paper preparation and study. EP provided new information necessary to revise the paper.

## Conflict of Interest Statement

The authors declare that the research was conducted in the absence of any commercial or financial relationships that could be construed as a potential conflict of interest.
